# Global evidence on the rapid adoption of telemedicine in primary care during the first 2 years of the COVID-19 pandemic: a scoping review protocol

**DOI:** 10.1186/s13643-022-01934-3

**Published:** 2022-06-19

**Authors:** Daniela Valdes, Lama Alqazlan, Rob Procter, Jeremy Dale

**Affiliations:** 1 Department of Computer Science, University of Warwick, Coventry, CV4 7AL UK; 2 Warwick Medical School, University of Warwick, Coventry, CV4 7AL UK

**Keywords:** Global Health, Pandemic response, Patient experience, Patient-clinician trust, Primary care, Telemedicine

## Abstract

**Background:**

Before the declaration of the COVID-19 pandemic in March 2020, primary care in most countries relied on face-to-face consultations, with relatively limited use of telemedicine. Lockdowns and social distancing measures during the early stages of the pandemic led to rapid, widely spread telemedicine adoption in healthcare settings. The rapid uptake that occurred following the onset of these pandemic-induced measures in countries such as the UK, Canada and New Zealand prompts questions around the drivers, extent and sustainability of this transformation in clinical practice at the global level, as the research in this area is still emerging. The purpose of this scoping review is to explore the global evidence surrounding the rapid adoption of telemedicine in primary care settings during the first 2 years of the COVID-19 pandemic through three lenses: patient experience, health inequalities and patient-clinician trust, with the purpose of identifying elements contributing to the sustainability of this innovation.

**Methods:**

A draft protocol was tested through an initial search on Ovid Medline, Web of Science and Google Scholar with additional searches on the Cochrane Database. This informed the final selection of terms which will be used to search Ovid, Web of Science, Google Scholar, PROSPERO, Cochrane Library and others, filtering for studies from the pandemic declaration onwards. Additional grey literature reports will be sourced through simplified searches on Google in widely spoken languages. Duplicates will be removed by screening titles. Abstracts and grey literature text extracts will be screened based on pre-set eligibility criteria by two researchers. Abstracts (and extracts in the case of grey literature) will be mapped against the domains of the Non-adoption, Abandonment, and challenges to Scale-up, Spread and Sustainability (NASSS) framework by two researchers. Data will be presented in table format.

**Discussion:**

This review will map the current literature to identify current gaps in evidence related to the adoption of telemedicine after the declaration of the pandemic in March 2020. The use of simplified searches in the several spoken languages in the world is aimed at capturing more immediate non-academic reflections and experiences on this major service change at a global level.

**Systematic review registration:**

The study has been registered on Open Science Framework and can be accessed through the following URL: https://osf.io/4z5ut/

## Background

Pre-pandemic evidence synthesis (published/submitted in late 2019) [1] identifies a number of ‘barriers’ or ‘objections’ that justify the low uptake of alternative consultation mediums. For instance, a rapid evidence synthesis on ‘digital-first’ primary care, confirmed that uptake of digital channels for patient’s first point of contact was low, and identified concerns around technology, workload and confidentiality  [2] as main barriers. A scoping review (focused on video consultations only) identified that this mode of delivery was not appropriate in many situations and as such face-to-face consultations were preferred [2, 3].

Following the WHO’s announcement on 11 March 2020 declaring COVID-19 a pandemic [4], the radical change seen during the first 2 years of the pandemic indicates how previously identified barriers and objections were rapidly overcome as shown in the below examples.

- In England, for example, data for older patients shows that the rate of remote consultations more than doubled between February and May 2020 [5], following a mandated move to total triage in the English National Health Service [6].
- In the Netherlands, the shift from mainly face to face to virtual consultations happened within a week, as part of the pandemic response plans [7].
- In the USA, the implementation of the ‘Expansion of telehealth with 1135 waiver’ [8] signalled a critical regulatory move for Medicaid Services, with a 154% increase in the year to March 2020 [9].

A preliminary search for existing systematic/scoping reviews on the topics of interest was conducted in PubMed (4 results) and PROSPERO (an international database of prospectively registered systematic reviews in health and other areas where there is a health-related outcome [10]—3 results) on the 13 January 2022 (see the Appendix for the search terms). We found four published reviews: one using the NASSS framework [11] to synthesised evidence around spread, scalability and adoption of videoconsulting in health care, albeit pre-pandemic [12]; virtual primary care in Australia [13]; opinions regarding the use of telemedicine by patients from populations during the pandemic [14]; implementation, adoption, and perceptions of Telemental Health during the pandemic [15]); and three ongoing reviews (on telemedicine interventions for type 2 diabetes in primary care [16]; and on factors affecting the uptake of remote consultations for long-term condition management in primary care [17]). The current version of the Cochrane library special collection on ‘Coronavirus (COVID-19): remote care through telehealth’ (last updated on 29 July 2021) only includes a review on healthworkers’ perceptions on telehealth in primary care [18]. Both PubMed and PROSPERO abstracts will be added to the screening pool.

### Rationale

Lockdowns and social distancing measures during the early stages of the pandemic led to rapid, widely spread telemedicine adoption in healthcare settings. The rapid uptake that occurred following the onset of these pandemic-induced measures in countries such as the UK, Canada and New Zealand prompts questions around the sustainability of this transformation in clinical practice at the global level particularly once lockdowns ease, as the research in this area is still emerging. It is important to understand what lies at the core of the rapid shift to telemedicine following years of limited penetration. In the UK, both the Health Foundation [19] and the UK Comptroller and Auditor General [20] issued recommendations of the need of compiling lessons around service shifts accelerated by the pandemic and digital transformation respectively. This understanding can help (i) address emerging concerns around potential disenfranchising of patients or particular population groups, given limited evidence on patient experience in this area and its potential to exacerbate health inequalities; (ii) evaluate the potential impacts on the patient-clinician relationship arising from teleconsultations and (iii) use these elements to support public discussions about the sustainability of these interventions in future healthcare delivery.

To support greater understanding in these areas, this scoping review explores the move to telemedicine in the context of primary care around the world during the first 2 years of the pandemic, with a focus on patient experience, health inequalities and patient-clinician trust. This comparative approach around digitisation builds upon recent academic literature in the area, which so far has focused on either the digital aspect [21] or pandemic response [7], mostly in English-speaking countries. This research will seek to incorporate academic and non-academic literature through other documents in widely spoken languages to capture more immediate responses, emerging voices and experiences worldwide [22]. The selection of a scoping review methodology responds to the identifying evidence in what is still an emerging field, as our focus is on post-pandemic induced changes.

## Key definitions and theoretical framework

### Telemedicine

The review will focus on the clinical practice of telemedicine (the interaction between patient and clinician). As such it draws on the World Health Organization’s definition of telemedicine [23].

The delivery of health care services, where distance is a critical factor, by all health care professionals using information and communication technologies for the exchange of valid information for diagnosis, treatment and prevention of disease and injuries […] all in the interests of advancing the health of individuals and their communities (World Health Organization, 2010, p9).

Telemedicine will be defined, in the negative, as those modalities of clinician/patient interaction that do not require physical presence of the individuals in the same premises (either a primary care clinic or the patient’s home). In the positive, these are modalities where the clinician/patient interaction is held or mediated in a ‘virtual’ (as opposed to physical, premises-based) environment, through the use of a particular telecommunication technology. This definition is closely aligned with that provided by Sood et al. [24] following a systematic review of over one hundred peer-reviewed perspectives. In all cases, the clinician is able to access and edit the patient’s record, and the appointment might be pre-scheduled, clinician-initiated or patient-initiated, synchronous or asynchronous.

These ‘virtual’ environments have been further described in Olayiwola et al., [25].

- Telephone visits: conversation happens over the telephone.
- Video visits: conducted through a (secure) video platform
- E-consultations: asynchronous discussions between clinician and patient, which are initiated by the patient through a patient portal which allows to specify their complaint. In some cases, the portals include a triaging protocol and allow to capture relevant details from the patient’s history.

In some settings, the terms ‘econsultations’ or ‘econsults’ can also apply to clinician/clinician discussions; but for the purposes of this review, these will be excluded.

### Primary care

Traditionally, primary care covers a multidisciplinary team of healthcare professionals dealing with areas related to communicable, non-communicable disease, prevention and management [26]. A standard definition is provided in the Alma Ata Declaration [27]:

…essential health care based on practical, scientifically sound and socially acceptable methods and technology made universally accessible to individuals and families in the community through their full participation and at a cost that the community and country can afford to maintain at every stage of their development in the spirit of self-reliance and self-determination. It forms an integral part both of the country’s health system, of which it is the central function and main focus, and of the overall social and economic development of the community. It is the first level of contact of individuals, the family and community with the national health system bringing health care as close as possible to where people live and work, and constitutes the first element of a continuing health care process.

The above definition also specifies this is a community-based (as opposed to a ‘hospital’/secondary care service). Hospital or secondary healthcare services, as well as educational activities, will be excluded from the review. Notwithstanding the multidisciplinary nature of primary care, searches will focus on the consultations of doctors and nurses as medical professionals.

### Population

The above definition of primary care also helps specify the population of interest, as healthcare services which are ‘universally accessible to individuals and families in the community’ [27]. No population exclusion will be applied during the screening process.

### Theoretical framework (NASSS framework)

The Non-adoption, Abandonment, and challenges to Scale-up, Spread and Sustainability (NASSS) framework [11] identifies six domains (and additional sub-domains) or lenses to examine adoption and critically, sustainability over time. These domains are the illness or condition, the technology, the value proposition, the adopter system, the organisations, the wider system and the seventh domain is the dynamic element—how these domains change over time. The framework evaluates how the simplicity or complexity of the transformation (acting over various domains) can either support spread and sustainability (in the case of simple transformations) or lead to abandonment or non-adoption (in the case of complex transformations in multiple domains).

This framework (co-developed by one of the co-authors of this review), has been selected because (i) it is evidence-based, (ii) focused on healthcare (as opposed to other more generic models of technology adoption), (iii) explores what happens ‘after’ adoption—and in our case has a specific domain focused on sustainability and (iv) because the multiple domains facilitate the mapping exercise of the findings, without the need to resort to further thematic analysis/grounded theory. The mapping will be undertaken by highlighting the various themes identified in the literature and comparing them to the relevant NASSS dimensions. For example, in the case of the US regulatory provision allowing for financial recognition of telemedicine appointments would be labelled as both a simplifying ‘wider system’ change but also as enabling change in the value proposition offered by the technology.

## Methods

The scoping review will be conducted in accordance with (i) the Joanna Briggs Institute (JBI) methodology [28], the latest update on the JBI methodological guidance [29], and the corresponding JBI evidence synthesis scoping review protocol template (Copyright © 2014, Aries Systems Corporation).

### Review questions and aims

What is the evidence available surrounding the rapid adoption of telemedicine in primary care settings worldwide during the first 2 years of the COVID-19 pandemic?

Specific objectives are to:

I. Understand the drivers behind the rapid adoption of telemedicine since the official declaration of COVID-19 as a pandemic in March 2020 as to identify elements that can support its sustainability beyond the pandemic;
II. Determine impacts of telemedicine adoption across countries across three specific domains: patient experience; health inequalities; patient-clinician trust;
III. Map the findings in the context of the NASSS framework [11] to identify gaps in evidence.

### Population-concept-context (PCC) summary

#### Population

The review will focus on primary care services offered to the general population. Studies focusing on specific population groups or those suffering from particular conditions within a particular country or geographical area will be included.

#### Concept

While the key concept under consideration is the adoption of ‘telemedicine’, as defined above, we will narrow our inclusion criteria on the sustainability of the interventions, patient experience, health inequalities and patient-clinician trust.

#### Context

The context is primary care services provided during the COVID-19 pandemic in any setting or country during the first 2 years of the pandemic.

### Design

Table 1 below summarises the inclusion and exclusion criteria.

**Table 1:** Inclusion and exclusion criteria

Category	Inclusion	Exclusion
Population	Primary care population or a population group/community within the primary care population by their age or ethnicity (such as children elderly, or indigenous people); or suffering from a particular condition (including case studies) Population groups defined by nature of a residential setting (prison population care/nursing home population).	
Healthcare setting	Primary care practice, primary care, family care, ambulatory/outpatient care	Other clinical settings (Secondary/tertiary inpatient, community). Within community exclude mental health, palliative care, physiotherapy, dental and pharmacy.
Context	First 2 years of the coronavirus pandemic at the global level (since March 2020–March 2022).	Telemedicine before the pandemic declaration. Telemedicine but not in the context of the pandemic
Concept	Telemedicine adoption, increased use or sustainability. Include patient surveys and patient experience, or impacts on health inequalities or patient-clinician trust Includes systematic and other reviews as well as observational studies. Include clinical trials over telemedicine systems if telemedicine is replacing a face to face appointment. Include protocols for remote clinical assessments	Exclude surveys or opinions on the idea of adoption. Exclude clinician to clinician consultations. Exclude other mobile health apps or medical apps or wider e-health studies. Exclude clinical trials if telemedicine is not used. Excludes health coaching or health advisory (by non-clinical volunteers.)

#### Types of sources

This scoping review will consider quantitative, qualitative and mixed methods study designs for inclusion. In addition, systematic reviews, protocols, other text and opinion papers will be considered for inclusion in the proposed scoping review.

#### Search strategy

The search strategy has been developed to ensure transparency and reproducibility and the Preferred Reporting Items for Systematic Reviews and Meta-analyses for Scoping Reviews (PRISMA-ScR) [30] checklist has been used to verify its appropriateness. Search terms around telemedicine, primary care and COVID-19 have been expanded by a limited search on Ovid Medline and Web of Science, Warwick Librarian advice (which helpfully identified COVID-19 search terms from NICE [31]), and a study of previous protocols in telemedicine in general practice [32] and Primary Care Cochrane Library Protocols. All identified search terms as well as examples of two the searches (for academic and grey literature) have been provided in the Appendix.

#### Special considerations for grey literature

Given the rapid developments associated with the implementation of telemedicine in primary care settings during the first 2 years of the pandemic, this scoping review considers emerging thinking by mapping ‘grey’, non-academic literature. Preference will be given to ‘first-tier’ grey literature, comprising of government policy and reports, as well as think-tank reports and white papers (mostly found in PDF format). This documentation is highly retrievable and credible [33, 34]. A similar methodology has been used in other reviews [35, 36].

Searching for grey literature, the use of other languages other than English and the focus in other countries is aimed at increasing representativeness (from a Global Health perspective), to capture non-academic voices and experiences [22]. We will use Google by selecting the first 30 results by relevance of PDF documents emerging from simplified search terms (telemedicine; ‘Primary Care’; COVID-19; patient experience; health inequalities; patient-clinician interaction) in the top languages in the world (the top five in terms of internet users—English, Chinese, Spanish, Arabic, Portuguese [24]—and the first language of the top five countries by population [37] Hindi, Urdu, Indonesian ). Mindful of the lack of representation of African countries in preliminary searches, we will undertake 5 additional google searches in English focusing on the five largest African countries by population (Nigeria, Ethiopia, Democratic Republic of Congo, Egypt and South Africa [38]), extracting the top ten results. Terms used for non-English searches were found using Machine Translation (Google Translate) and discussions with native speakers. The list of terms is found in the Appendix.

Abstracts and excerpts for searches in other languages will focus on PDF documents published between March 2020 and the date of the search to focus on the emerging evidence surrounding the early stages of the coronavirus pandemic period only. These will be translated using Machine Translation if no abstract in English is available [22]. In case the grey literature does not include an abstract or summary, researchers will extract up to four paragraphs for screening and mapping: the first full paragraph where ‘telemedicine’ term is found; and three other paragraphs when either of the three expressions are found (patient experience, health inequalities and patient-clinician trust). These paragraphs will be translated with automated translation or by native speakers when possible.

Final searches will be undertaken between February and March 2022, including academic databases such as EMBASE, LIVIVO, SCOPUS, Ovid Medline, Web of Science, Scielo and Google Scholar (first 30 results by relevance), as well as PROSPERO results. No language restrictions will be included in the academic searches.

### Evidence selection

An initial search on Ovid Medline, Web of Science and Google Scholar was undertaken in December 2020. Two researchers with different academic backgrounds (healthcare management and computer science) independently screened all abstracts to (i) refine search terms [32], (ii) test the protocols’ inclusion criteria and (iii) train a prioritisation tool [39]. This ‘pilot’ process helped refine both the search and the screening parts of the review.

The abstracts and excerpts across all languages of the final search results will be uploaded to the automated tool for prioritisation and final screening by two researchers. Reasons for exclusion of evidence that does not meet the inclusion criteria will be recorded and reported in the scoping review.

As a final part of the search strategy, the lead researcher will undertake forward and backward reference searches to identify any other potential studies that might have been missed in the search process.

### Data extraction

Data will be extracted by two researchers. Data extraction from the full-text selected academic documents using an Excel/NVIVO table template as a data extraction tool (for grey literature, the extraction will focus on the selected excerpts only). The template will include headings such as author, title, year, abstract, type of document, population, concept, context, methods, key findings relevant to review questions and the country of study. These elements will be mapped in tabular form against the NASSS framework [11] domains on the NVIVO computer software (version 12 © QSR International 2020). It is expected that some of the studies will touch upon one or more categories of the framework.

Any modifications to the protocol will be reported in full in the scoping review.

### Data analysis and presentation

Data will be analysed by two researchers. In agreement with the latest JBI methodological guidance [28] [29], no critical appraisal will be undertaken, and the final presentation of results will consist of two sections:

1. In the first section, the results of the search will be presented in a Preferred Reporting Items for Systematic Reviews and Meta-analyses for Scoping Reviews (PRISMA-ScR) flow diagram [24]
2. In the second section, the elements of the PCC inclusion criteria will be used to provide a narrative summary of the results and describe how they relate to the review objective and question. The authors will also include a mapping in tabular form against the NASSS framework [11] of the extracted data alongside a basic descriptive analysis of the frequency of counts of the data extracted (populations, concept, context and the country of origin) and type of document identified.

## Discussion

This session briefly outlines the strengths and limitations of the research design.

### Strengths

The JBI methodology [28, 29] for scoping reviews is recognised as appropriate in the context of the identification of literature gaps. Having a second researcher independently testing inclusion and exclusion criteria provides robustness to the training of the prioritisation tool and can limit bias. The use of multi-language searches for grey literature is used to amplify the voices of non-English-speaking countries and experiences. Further, using the NASSS framework [11] to map the literature is an innovative approach to the scope as it provides a tried and tested framework of the study of technological transformation in healthcare.

### Limitations

It is recognised that this is an emerging field in the literature and as such, there is limited academic output at this stage that has been through peer review. Further review might be beneficial in a few years to capture new published academic research in this area. There are limitations in the reproducibility of google searches given that search results are regularly updated depending on the popularity of individual results over time. To mitigate known limitations in comprehensibility and usability of Machine Translation of abstracts and excerpts [39, 40], the researchers have sought support as appropriate from native language speakers whenever feasible. While focusing on the increased use of telemedicine triggered by the COVID-19 pandemic, additional research is recommended to identify differences in drivers with other countries with long-standing experience in telemedicine (such as Norway).

## Data Availability

Not applicable. No datasets are currently available.

## Appendix

### Search terms and examples

#### PubMed Search

(((primary care) AND (telemedicine adoption) AND (pandemic)) AND ((patient experience) OR (health inequalities) or (patient clinician relationship))) AND ((systematic review[Title]) OR (scoping review[Title]))

#### PROSPERO SEARCH

(telemedicine AND (primary care)) AND (((coronavirus or corona-virus) AND (wuhan or beijing or shanghai or Italy or South-Korea or korea or China or Chinese or 2019-nCoV or nCoV or COVID-19 or Covid19 or SARS-CoV* or SARSCov2 or ncov)) OR (pneumonia AND Wuhan) or “COVID-19” or “2019-nCoV” or “SARS-CoV” or SARSCOV2 or 2019-nCov or “2019 coronavirus” or “2019 corona virus” or covid19 or ncov OR “novel corona virus” or “new corona virus” or “nouveau corona virus” or “2019 corona virus” OR “novel coronavirus” or “new coronavirus” or “nouveau coronavirus” or “2019 coronavirus”) AND (general_interest OR Health inequalities/health equity OR International development OR Public health including social determinants of health):HA NOT Animal:DB.

#### Simplified search terms for other languages

Top five internet languages

- English: telemedicine, “primary care”, “patient experience”, “health inequalities”, “patient-clinician trust”
- Chinese (simplified): 远程医疗, 初级保健, 患者体验,
- Spanish: Telemedicina, “atencion primaria”, “experiencia del paciente”, “inequidad en salud”, “confianza en el profesional de la salud”
- Arabic: “عدم المساواة الصحية “،”التطبيب عن بعد “, “الرعاية الصحية الأولية  “, ”تجربة المريض   “, “القة بين الطبيب و المريض”
- Portuguese (Brazil) Telemedicina, “atenção primária a saúde”, “experiência do paciente”, “desigualdade na saúde”, “confiança entre paciente e medico”

First language spoken in the most populated countries in the world (if not in the above list)

- Hindi (India): टेलीमेडिसिन, पारिवारिक चिकित्सा, “रोगी अनुभव”, “स्वास्थ्य असमानता”, “रोगी और चिकित्सक के बीच विश्वास”
- Indonesian: telemedicine, “kedokteran keluarga”, “pengalaman pasien”, “ketimpangan Kesehatan”, “kepercayaan antara pasien dan dokter”
- Urdu (Pakistan):” مریض اور معالج کے درمیان اعتماد ”ٹیلی میڈیسن” “ ,“بنیادی دیکھ بھال”, مریض کا تجربہ”, “صحت کی عدم مساوات”,

#### OVID (Medline all) - Search conducted on 13 January 2022

**Table:**

Search	Query (title, abstract, keyword heading)	Records retrieved
#1	telemedicine or telehealth or “digital health” or phone or telephone or video or virtual or remote or e-consults or e-consultation or tele-consult or phone consult or ehealth or tele-health or tele-medicine or e-health	371,957
#2 [31]	coronavirus* or coronovirus* or coronavirinae* or Coronavirus* or Coronovirus* or Wuhan* or Hubei* or Huanan or “2019-nCoV” or 2019nCoV or nCoV2019 or “nCoV-2019” or “COVID-19” or COVID19 or “CORVID-19” or CORVID19 or “WN-CoV” or WNCoV or “HCoV-19” or HCoV19 or CoV or “2019 novel*” or Ncov or “n-cov” or “SARS-CoV-2” or “SARSCoV-2” or “SARSCoV2” or “SARS-CoV2” or SARSCov19 or “SARS-Cov19” or “SARSCov-19” or “SARS-Cov-19” or Ncovor or Ncorona* or Ncorono* or NcovWuhan* or NcovHubei* or NcovChina* or NcovChinese* or pandemic or outbreak or epidemic	398,362
#3	“general practice” or “general practitioner” or “general physician” or “general clinician” or “general doctor” or “general nurse” or “general nursing” or “general medicine” or “family practice” or “family practitioner” or “family physician” or “family clinician” or “family doctor” or “family nurse” or “family nursing” or “family medicine” or “Primary Care clinic” or “primary care practitioner” or “primary care physician” or “primary care clinician” or “primary care doctor” or “primary care nurse” or “primary care nursing” or “primary care medicine” or “Primary health care clinic” or “primary health care practitioner” or “primary health care physician” or “primary health care clinician” or “primary health care doctor” or “primary health care nurse” or “primary health care nursing” or “primary health care medicine” or “primary healthcare clinic” or “primary healthcare practitioner” or “primary healthcare physician” or “primary healthcare clinician” or “primary healthcare doctor” or “primary healthcare nurse” or “primary healthcare nursing” or “primary healthcare medicine” or GP or doctor or nurse or physician or clinician	100,210
#4	#1 AND #2 AND 3	318
#5 [14]	“patient experience” or “patient satisfaction” or “patient opinion” or “PREM”, or “patient survey” or “patient preference” or “client experience” or “client preference” or “service user preference” or “client experience survey” or “healthcare survey” or “healthcare evaluation” or “health surveys” or questionnaire or survey or “patient voice” or “service user voice” or “patient perspective” or “patient centered quality” or “patient perception” or “patient view”	1,019,204
#6 [14]	“Health inequalities” or “health inequality” or “health inequity” or “health inequities” or “socioeconomic determinants” or “social determinants of health” or “social disparities” or “vulnerable population” or “disadvantaged populations” or “low income populations” or “ethnic minority” or “indigenous populations” or “underserved populations” or “health inequities” or “geographical inequalities” or “remote populations” or “deprived community” or “social deprivation” or “protected characteristics” or gender, age, “maternity status” or LGBTQ+ or veteran or transgender or “minoritised populations” or disabled or “learning disability” or “married” or “religious populations” or refugee or migrant	134,379
#7	“patient trust” or “trust in clinician” or “trust in physician” or “trust in nurse” or “trust in medical professionals” “trust in health practitioner” or “trust in medical profession” or “trust in medical decision making” or “physician-patient Relationship” or “patient-clinician relationship” or “patient clinician communication” or “patient clinician trust” or “patient clinician rapport” or “patient clinician relationship” or “physician patient relationship” or “patient rapport” or “rapport with patient” or “doctor patient relationship”, “clinician patient relationship” or “nurse patient relationship” or “doctor patient rapport” or “patient clinician interaction” or “patient clinician encounter” or trust or reliance or confidence	640,538
#8	#5 OR #6 OR #7	1,677,187
#9	#4 AND #8	128
Limited to 2020 onwards		116

#### Google Spanish

telemedicina pandemia “experiencia del paciente” OR “inequidad en salud” OR “confianza en el profesional de la salud” “atencion primaria” filetype:pdf

Advanced Search

*Find pages with...*

all these words: telemedicina COVID-19

this exact word or phrase: “atencion primaria”

any of these words: “experiencia del paciente” OR “inequidad en salud” OR “confianza en el profesional de la salud”

*Then narrow your results by...*

language: Spanish

file type: Adobe Acrobat PDF

Date: From 3/11/2020 onwards

*Results: Provide 30 results per page*

*Results*

Over five pages of results, first 30 by relevance to be selected.

## Abbreviations

JBI: Joanna Briggs Institute
NASSS: Non-adoption, Abandonment, and challenges to Scale-up, Spread and Sustainability
PCC: Population, concept, context
PRISMA-ScR: Preferred Reporting Items for Systematic Reviews and Meta-Analyses Extension for Scoping Reviews

## References

[1] Natalie Robson, Hassan Hosseinzadeh. How does the use of Telehealth compare to the face- to- face delivery of care in relation to the management of adults living with type 2 diabetes? A systematic review and meta-analysis of randomised controlled trials. [cited 2022 Jan 13]; Accessed 13 January 2022 Available from: https://www.crd.york.ac.uk/PROSPERO/display_record.php?RecordID=250847

[2] Rodgers M, Raine G, Thomas S, Harden M, Eastwood A (2019). Informing NHS policy in ‘digital-first primary care’: a rapid evidence synthesis. Health Serv Deliv Res..

[3] Thiyagarajan A, Grant C, Griffiths F, Atherton H. Exploring patients’ and clinicians’ experiences of video consultations in primary care: a systematic scoping review. BJGP Open. 2020;4:bjgpopen20X101020. 10.3399/bjgpopen20X101020 PMC7330183 32184212

[4] WHO Director-General’s opening remarks at the media briefing on COVID-19 - 11 March 2020. [cited 2021 Feb 20]. Available from: https://www.who.int/director-general/speeches/detail/who-director-general-s-opening-remarks-at-the-media-briefing-on-covid-19%2D%2D-11-march-2020

[5] Joy M, McGagh D, Jones N, Liyanage H, Sherlock J, Parimalanathan V (2020). Reorganisation of primary care for older adults during COVID-19: a cross-sectional database study in the UK. Br J Gen Pract..

[6] Guidance and Standard operating procedures. General practice in the context of coronavirus (COVID-19) Version 3.4. NHS England; 2020. Accessed February 6, 2021. Available from: https://www.england.nhs.uk/coronavirus/publication/managing-coronavirus-covid-19-in-general-practice-sop/

[7] Huston P, Campbell J, Russell G, Goodyear-Smith F, Phillips RL, Weel C van, et al. COVID-19 and primary care in six countries. BJGP Open. Royal College of General Practitioners; 2020;4. [cited 2020 Nov 12] Available from: https://bjgpopen.org/content/4/4/bjgpopen20X101128 10.3399/bjgpopen20X101128 PMC7606153 32900708

[8] Centers for Medicare & Medicaid Services. Medicare Telemedicine Health Care Provider Fact Sheet. 2020 [cited 2020 Nov 29]. Available from: https://www.cms.gov/newsroom/fact-sheets/medicare-telemedicine-health-care-provider-fact-sheet

[9] Koonin LM. Trends in the Use of Telehealth During the Emergence of the COVID-19 Pandemic — United States, January–March 2020. MMWR Morb Mortal Wkly Rep. 2020;69. [cited 2020 Dec 2] Available from: https://www.cdc.gov/mmwr/volumes/69/wr/mm6943a3.htm 10.15585/mmwr.mm6943a3 PMC7641006 33119561

[10] PROSPERO. [cited 2021 Feb 28]. Available from: https://www.crd.york.ac.uk/PROSPERO/#aboutpage

[11] Greenhalgh T, Wherton J, Papoutsi C, Lynch J, Hughes G, A’Court C (2017). Beyond Adoption: A New Framework for Theorizing and Evaluating Nonadoption, Abandonment, and Challenges to the Scale-Up, Spread, and Sustainability of Health and Care Technologies. J Med Internet Res..

[12] Jonnagaddala J, Godinho MA, Liaw S-T. From telehealth to virtual primary care in Australia? A Rapid Scoping Review. Int J Med Inf. 2021;104470. 10.1016/j.ijmedinf.2021.104470 PMC9761082 34000481

[13] James HM, Papoutsi C, Wherton J, Greenhalgh T, Shaw SE. Spread, scale-up, and sustainability of video consulting in health care: systematic review and synthesis guided by the NASSS framework. J Med Internet Res. 2021;23:e23775. 10.2196/23775 PMC7837451 33434141

[14] Silva AB, Sindico SRF, Carneiro AC, Henrique SM, Fernandes AG, Gomes JP, et al. COVID-19 remote consultation services and population in health inequity-concentrating territories: a scoping review. Telemed E-Health. 2021;27:881–97. 10.1089/tmj.2021.0145 PMC8380793 34232749

[15] Appleton R, Williams J, Juan NVS, Needle JJ, Schlief M, Jordan H, et al. Implementation, adoption, and perceptions of telemental health during the COVID-19 pandemic: systematic review. J Med Internet Res. 2021;23:e31746. 10.2196/31746 PMC8664153 34709179

[16] Ibsen D, Mba C, Vounzoulaki E, Cerullo E, Torres H, Olsson K, et al. Effectiveness of telemedicine in prevention of type 2 diabetes: a systematic review and meta-analysis of interventions relevant for primary care settings. [cited 2022 Jan 13]; Available from: https://www.crd.york.ac.uk/PROSPERO/display_record.php?RecordID=210829

[17] Scott E, Finney A, Swaithes L, Wynne-Jones G. Factors affecting uptake and delivery of video group consultations for the management of long-term conditions in primary care general practice: a systematic review. [cited 2022 Jan 13]; Available from: https://www.crd.york.ac.uk/PROSPERO/display_record.php?RecordID=220258

[18] Gleeson C, Lasserson MKT, Featherstone R, Mehta Declan Devane M. Coronavirus (COVID-19): remote care through telehealth. 2020 [cited 2020 Dec 23]; Available from: https://www.cochranelibrary.com/collections/doi/SC000043/full

[19] Lewis R, Pereira P, Thorlby R, Warburton W. Understanding and sustaining the health care service shifts accelerated by COVID-19. 2020;17.

[20] UK National Audit Office. Reportby the Comptroller and Auditor General - Digital transformation in the NHS. 2020 p. 57.

[21] Varsamis D (2020). Incentives and levers for digitising and integrating primary care in New Zealand, Australia and the USA.

[22] Walpole SC (2019). Including papers in languages other than English in systematic reviews: important, feasible, yet often omitted. J Clin Epidemiol..

[23] World Health Organization, editor. Telemedicine: opportunities and developments in member states: report on the second Global survey on eHealth. Geneva: World Health Organization; 2010.

[24] Sood S, Mbarika V, Jugoo S, Dookhy R, Doarn CR, Prakash N, et al. What Is Telemedicine? A Collection of 104 Peer-Reviewed Perspectives and Theoretical Underpinnings. https://home.liebertpub.com/tmj. Mary Ann Liebert, Inc. 2 Madison Avenue Larchmont, NY 10538 USA; 2007 [cited 2021 Feb 28]. Available from: https://www.liebertpub.com/doi/abs/10.1089/tmj.2006.0073 10.1089/tmj.2006.0073 17999619

[25] Olayiwola JN, Magaña C, Harmon A, Nair S, Esposito E, Harsh C (2020). Telehealth as a Bright Spot of the COVID-19 Pandemic: Recommendations From the Virtual Frontlines (“Frontweb”). JMIR Public Health Surveill..

[26] Beks H, Ewing G, Muir R, Charles J, Paradies Y, Clark R (2020). Mobile primary health care clinics for Indigenous populations in Australia, Canada, New Zealand and the United States: a scoping review protocol. JBI Evid Synth..

[27] World Health Organisation. Declaration of Alma-Ata. 1978. Available from: https://www.euro.who.int/__data/assets/pdf_file/0009/113877/E93944.pdf. Accessed 6 Feb 2022.

[28] Aromataris E, Munn Z, editors. JBI Manual for Evidence Synthesis. JBI; 2020 [cited 2020 Oct 15]. Available from: https://wiki.jbi.global/display/MANUAL

[29] Peters MDJ, Marnie C, Tricco AC, Pollock D, Munn Z, Alexander L (2020). Updated methodological guidance for the conduct of scoping reviews. JBI Evid Synth..

[30] Tricco AC, Lillie E, Zarin W, O’Brien KK, Colquhoun H, Levac D, et al. PRISMA Extension for Scoping Reviews (PRISMA-ScR): Checklist and Explanation. Ann Intern Med. Am Coll Physicians; 2018;169:467–73. 10.7326/M18-0850 30178033

[31] NICE. Interim process and methods for developing rapid guidelines on COVID-19. NICE; 2020 p. 12. Available from: https://www.nice.org.uk/process/pmg35. Accessed 20 Apr 2022.

[32] Downes MJ, Mervin MC, Byrnes JM, Scuffham PA (2015). Telemedicine for general practice: a systematic review protocol. Syst Rev..

[33] Adams RJ, Smart P, Huff AS (2017). Shades of Grey: Guidelines for Working with the Grey Literature in Systematic Reviews for Management and Organizational Studies. Int J Manag Rev..

[34] Tyndall J (2008). How low can you go? Toward a hierarchy of Grey Literature.

[35] Engels N, de Graav G, van der Nat P, van den Dorpel M, Bos WJ, Stiggelbout AM (2020). Shared decision-making in advanced kidney disease: a scoping review protocol. BMJ Open..

[36] Milne-Ives M, Lam C, van Velthoven M, Meinert E (2020). The Impact of Brexit on the Pharmaceutical Supply Chain of the United Kingdom: Scoping Review Protocol. JMIR Res Protoc.

[37] Top Ten Internet Languages in The World - Internet Statistics. [cited 2021 Jan 17]. Available from: https://www.internetworldstats.com/stats7.htm

[38] Population, total | Data. [cited 2021 Jan 17]. Available from: https://data.worldbank.org/indicator/SP.POP.TOTL?most_recent_value_desc=true

[39] Ouzzani M, Hammady H, Fedorowicz Z, Elmagarmid A (2016). Rayyan-a web and mobile app for systematic reviews. Syst Rev..

[40] Tongpoon-Patanasorn A, Griffith K (2020). Google Translate and Translation Quality: A Case of Translating Academic Abstracts from Thai to English. PASAA J Lang Teach Learn Thail. Chulalongkorn University Language Institute.

